# Chloramine

**Published:** 1916-10

**Authors:** 


**Chloramine:** Preparation, Properties and Use. Dakin, Cohen and Kenyon, Brit. Med. Jour., Jan. 26. This is paratoluene-sodium-sulphochloramid but as chloramine has been recorded in the U. S. as the name of an entirely dissimilar proprietary product, what the authors thus designate is marketed in the U. S. by the Abbott Laboratories, under the name CHLORAZENE. It is a white crystalline substance, with 3 molecules of water, soluble in water at ordinary temperatures up to 15% and in boiling water up to 50%. It is stable, non-corrosive even in concentrated solution, does not coagulate proteins and is practically non-toxic to animals, rabbits and guinea pigs tolerating 1 gram per kilo subcuteneously with no symptoms beyond moderate local reaction. As an antiseptic, it is closely comparable with sodium hypochlorite, weight for weight, its molecule being four times as heavy as the latter. It is much less irritating than sodium hypochlorite, as well as stable. The following table of comparative efficiency is given by the authors:

Two drops of a fresh culture of the organisms were suspended in 5 c. m. of fluid, either water or 50-per cent horse serum, and the antiseptic was allowed to act two hours at room temperature. In comparison, a few figures for sodium hypochlorite and phenol are added.

| | Chloramine | Sodium Hypochlorite | Phenol |
|---|---|---|---|
| Staphylococci in water | 1 : 500,000— | 1 : 500,000— | 1 : 250— |
| | 1 : 1,000,000+ | 1 : 1,000,000+ | 1 : 500+ |
| Staphylococci in | 1 : 1500— | 1 : 1500— | 1 : 50— |
| serum | 1 : 2500+ | 1 : 2000+ | 1 : 100+ |
| B. pyocyaneus in water | 1 : 200,000— | 1 : 100,000— | 1 : 200— |
| | 1 : 400,000+ | 1 : 1,000,000+ | 1 : 400+ |
| B. pyocyaneus in | 1 : 1250— | 1 : 2500— | 1 : 25— |
| serum | 1 : 2000+ | 1 : 5000+ | 1 : 50+ |
| Streptococci in water | 1 : 1,000,000— | | |
| Streptococci in serum | 1 : 2500— | | |
| | 1 : 5000+ | | |
| B. capsulatus in water | 1 : 10,000,000— | | |
| B. capsulatus in serum | 1 : 2500— | | |
| | 1 : 5000+ | | |

Complete sterilization is indicated by — while + indicates that organisms survived.

